# Independently Tunable Multipurpose Absorber with Single Layer of Metal-Graphene Metamaterials

**DOI:** 10.3390/ma14020284

**Published:** 2021-01-08

**Authors:** Chen Han, Renbin Zhong, Zekun Liang, Long Yang, Zheng Fang, Yiqing Wang, Anchen Ma, Zhenhua Wu, Min Hu, Diwei Liu, Shenggang Liu

**Affiliations:** 1 Terahertz Research Centre, School of Electronics Science and Engineering, University of Electronic Science and Technology of China, Chengdu 610054, China; 201721040324@std.uestc.edu.cn (C.H.); 201922021633@std.uestc.edu.cn (Z.L.); 201821020639@std.uestc.edu.cn (L.Y.); fangzhengfz@std.uestc.edu.cn (Z.F.); 201822021601@std.uestc.edu.cn (Y.W.); 201822021602@std.uestc.edu.cn (A.M.); wuzhenhua@uestc.edu.cn (Z.W.); hu_m@uestc.edu.cn (M.H.); dwliu@uestc.edu.cn (D.L.); liusg@uestc.edu.cn (S.L.); 2 Cooperative Innovation Center of Terahertz Science, University of Electronic Science and Technology of China, Chengdu 610054, China

**Keywords:** independently tunable, graphene, metamaterial absorber, single layer

## Abstract

This paper reports an independently tunable graphene-based metamaterial absorber (GMA) designed by etching two cascaded resonators with dissimilar sizes in the unit cell. Two perfect absorption peaks were obtained at 6.94 and 10.68 μm with simple single-layer metal-graphene metamaterials; the peaks show absorption values higher than 99%. The mechanism of absorption was analyzed theoretically. The independent tunability of the metamaterial absorber (MA) was realized by varying the Fermi level of graphene under a set of resonators. Furthermore, multi-band and wide-band absorption were observed by the proposed structure upon increasing the number of resonators and resizing them in the unit cell. The obtained results demonstrate the multipurpose performance of this type of absorber and indicate its potential application in diverse applications, such as solar energy harvesting and thermal absorbing.

## 1. Introduction

Metamaterials are a type of artificially engineered electromagnetic material composed of periodically arranged sub-wavelength elements. They exhibit excellent properties and applications such as negative refractive index [1], optical stealth [2], perfect lens imaging [3], and perfect optical absorption [4]. Metamaterial absorbers (MAs) have long been considered as an attractive candidate for optical applications and have been used for micro-bolometers, photodetectors, and invisible clocks [5,6,7]. The first conventional MA designed by Landy consisted of a dielectric layer placed between metallic resonators on the top and a gold substrate at the bottom [4]. Thus far, a variety of structures with different shapes, such as rectangular [8], split ring [9], concentric square rings [10], and their composites [11], have been utilized as resonators for MAs. Nevertheless, they are disadvantageous in that as long as the size of the resonators is fixed, the resonant frequencies remain unchanged for the above resonators. To ensure that the optical spectrum can be dynamically tuned, researchers have combined MAs with tunable materials such as liquid crystals [12], semiconductor silicon [13], and diodes [14]. Among them, graphene is a typical two-dimensional material which is expected to meet the requirements of tunable MAs. It is worth mentioning that the surface conductivity of graphene can be tuned by changing the Fermi level via electrostatic gating [15] or chemical doping [16,17], which is convenient for designing tunable components. Due to the unique properties of graphene, some dynamically tunable graphene-based absorbing structures have been reported, such as graphene disks [18], unbroken graphene [19], and a square graphene split ring [20]. Previous studies have found that multilayer metamaterials can exhibit multiple absorption bands, and the peaks can be tuned independently by adjusting the parameters of a certain layer, but this will require a more complex design and increase fabrication difficulties [21,22,23]. Achieving dynamically and independently tunable graphene-based metamaterial absorbers (GMAs) with a simple design to improve the performance of energy-absorbing structures has been one of the most important research topics in the recent past.

In this paper, an independently tunable multipurpose single-layer GMA is proposed at mid-infrared frequencies. Two perfect absorption peaks are obtained; each resonant frequency can be independently tuned with a high absorption rate of 99% by varying the Fermi level of graphene under a set of resonators. The absorber remains effective over a wide range of angles under oblique incidence. In addition, multi-band and broadband absorption are observed when appropriately sized resonators are etched in the unit cell. All these results indicate that the proposed structure can be widely used in solar ray harvesting and thermal absorbing.

## 2. Design and Simulation

**Figure 1:**
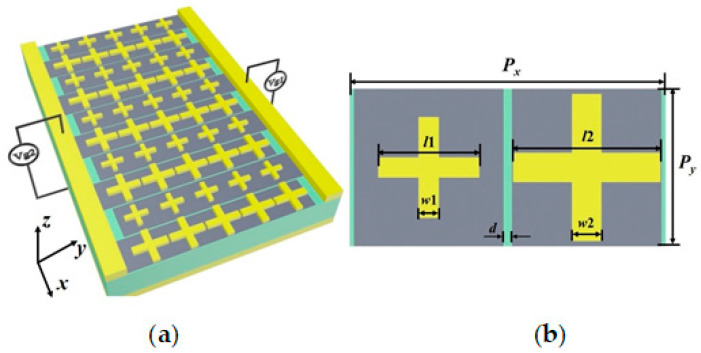
(**a**) Schematic diagram of the independently tunable multipurpose absorber with a single layer of metal-graphene metamaterials; (**b**) Top view of the unit cell.

A schematic diagram of the proposed GMA is illustrated in Figure 1a. The periodic arrays of metallic resonators, graphene ribbons, the insulated dielectric layer, and gold substrate are tightly stacked from top to bottom. The metallic pads on the plane of the graphene and the gold substrate act as electrodes where the gate voltages are applied. Figure 1b shows the geometric design of the resonators in the unit cell. It consists of two gold crosses of different dimensions. Graphene ribbons are placed under both the crosses, which are separated from each other by a small gap of *d* = 0.2 μm. Gate voltages *V*_g1_ and *V*_g2_ are applied to the two sets of graphene ribbons, and their Fermi levels are *E_f_*_1_ and *E_f_*_2_, respectively. The lengths of the resonator strips are *l*1 = 2.4 μm and *l*2 = 3.5 μm, and their widths are *w*1 = 0.48 μm and *w*2 = 0.7 μm. The periods of the unit cell are *P_x_* = 7.5 μm along the *x* direction and *P_y_* = 3.75 μm along the *y* direction. The dielectric layer has a thickness of 0.33 μm and comprises polytetrafluoroethylene with a relative permittivity of 2. The thickness of the ground plane is 0.3 μm, which is greater than its skin depth, ensuring that the transmission of electromagnetic waves can be completely suppressed. The relative permittivity of gold is defined as [24]:

(1) $$\varepsilon_{Au}=1-\frac{\omega_{p}^{2}}{\omega \left(\omega +j\gamma_{0}\right)}$$

Here, ω*_p_* = 1.37 × 10^16^ s^−1^ is the bulk plasmon frequency and γ_0_ = 4.08 × 10^13^ s^−1^ is the collision frequency.

The surface conductivity of graphene, *σ*_g_, can be determined by the Kubo formula [25,26]; it consists of intraband and interband contributions. In the mid-infrared region, the Fermi level is larger than half of the photon energy (*E_f_* > *ħω*/2), and the intraband contribution dominates the surface conductivity of graphene, while the interband transitions are negligible owing to Pauli blocking [27]. Consequently, it can be expressed by the following formula:

(2) $$\sigma_{g}=\sigma_{intra}\left(\omega ,\Gamma ,\mu_{c}\right)=\;j\frac{e^{2}k_{B}T}{\pi {ћ}^{2}\left(\omega +j\Gamma \right)}\left[\frac{\mu_{c}}{k_{B}T}+2ln\left(exp\left(-\frac{\mu_{c}}{k_{B}T}\right)+1\right)\right]$$

Here, *e* is the elementary charge, *k_B_* is the Boltzmann constant, *T* = 300 K is the Kelvin temperature, *ћ* = *h*/2π is the reduced Planck’s constant, and *ω* is the applied angular frequency. *Γ* is the carrier scattering rate, which is kept constant at 2.4 THz for this study. *μ_c_* is the chemical potential, which is equal to the Fermi level of graphene when *µ_c_* > *k_B_*.

**Figure 2:**
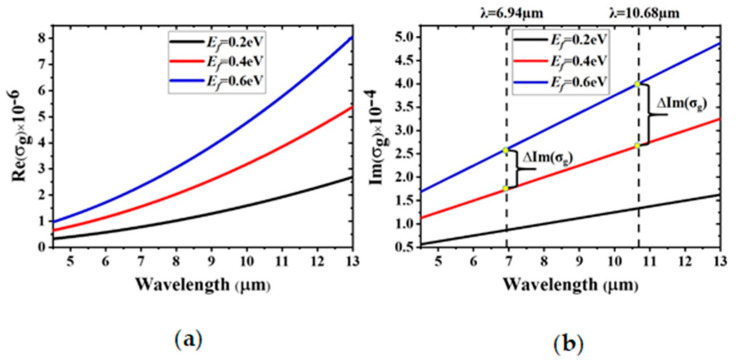
Changes in the surface conductivity of graphene with various Fermi levels at mid-infrared frequencies: (**a**) real part Re (*σ*_g_) and (**b**) imaginary part Im (*σ*_g_).

Figure 2 shows the variation in the real and imaginary parts of *σ*_g_ as the Fermi level changes from 0.2 to 0.6 eV. It is evident that *σ*_g_ can be dynamically tuned by changing the Fermi level, which is highly convenient for designing tunable absorbers working within a specific frequency range. According to the Maxwell equations, Re (*σ*_g_) is proportional to Im (*ε*_g_), which represents the absorption losses in graphene [20]. Thus, the amplitude modulation of the absorption peak is determined by Re (*σ*_g_) when *E_f_* is changed. In addition, the spectral tuning can be measured by ∆Im (*σ*_g_), which is explained in detail below.

According to the theory of multiple reflection and interference of the incident electromagnetic field, the absorption A (λ) can be obtained by A (λ) = 1 − R (λ) − T(λ), where R (λ) is the reflection and T (λ) is the transmission [28]. Since the gold substrate at the bottom is thicker than the skin depth, the transmittance is zero; thus, the absorption becomes A (λ) = 1 − R (λ).

## 3. Results and Discussion

**Figure 3:**
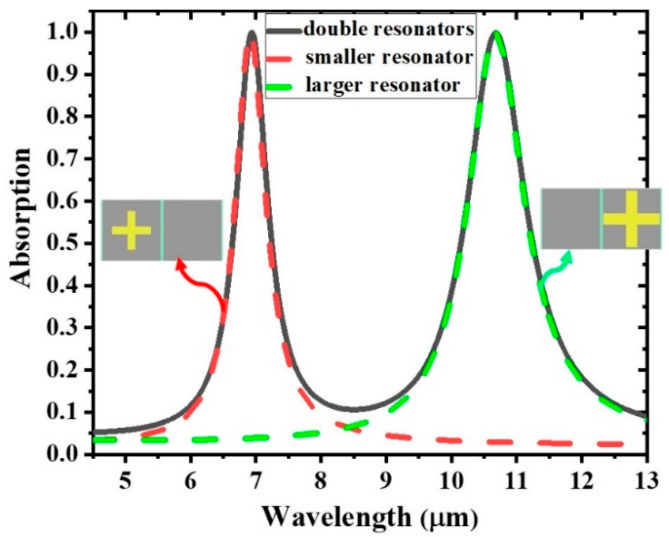
Absorption spectrum for absorbers constructed by double cross-shaped gold resonators (solid line) and single cross-shaped gold resonators (dashed lines).

Theoretical research on the proposed GMA was carried out using the finite elements solver COMSOL Multiphysics. When an s-polarized plane electromagnetic wave was incident on the double cross resonators, the absorption spectrum depicted by the solid curve in Figure 3 was obtained. Two absorption peaks greater than 99% are observed at 6.94 and 10.68 μm. When compared to the absorption peak of the single cross resonator, it is evident that the two absorption peaks originate from the two individual cross-shaped metallic resonators. The total absorbed energy efficiency is the integration of absorption A (λ) over the total incident energy at the regarded energy band, that is $E=\int_{\lambda_{min}}^{\lambda_{max}}A\left(\lambda \right)d\lambda /\left(\lambda_{max}-\lambda_{min}\right)$, which presents the overall capability of light conversion [29]. Here, λ*_min_* and λ*_max_* are the value of minimum and maximum wavelengths, respectively. Within the wavelength range from 4.5 to 13 μm, the value of *E* is 28.99% for the dual-band absorption structure. A larger value of absorption efficiency can be achieved via further reducing the wavelength range; the calculated *E* is 41.03% when the absorber works in the range of 6.5 to 11.5 μm. It should be noted that this range includes a large proportion of the atmospheric window which is the wave band with relatively high transmittance. The calculation means that nearly half of the incident light within the considered range could be absorbed. Therefore, the absorbers proposed in our research could acquire more of the incident energy, which will benefit the applications for light harvesting and thermal absorbing.

**Figure 4:**
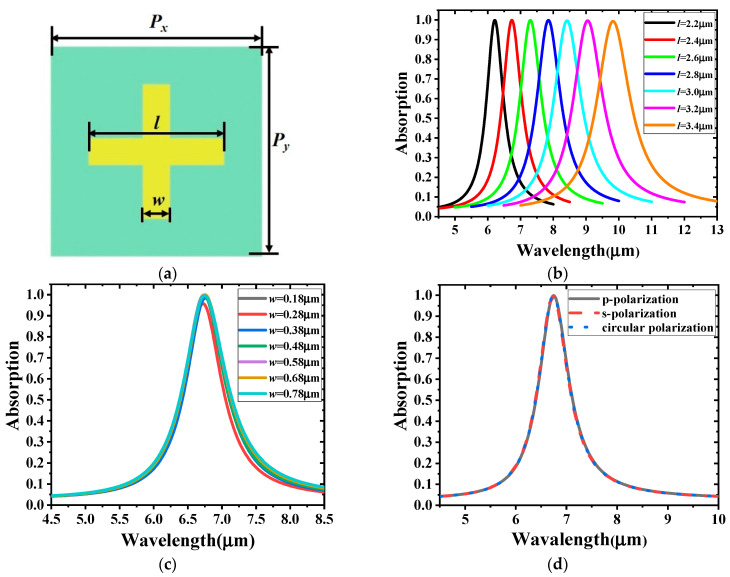
(**a**) Unit cell of the single-band absorber with *l* = 2.4 μm, *w* = 0.48 μm, and *P_x_* = *P_y_* = 3.75 μm; Simulated absorption spectra for different (**b**) lengths and (**c**) widths of the resonator strips; (**d**) Absorption curves for different polarizations of the incident wave.

To further demonstrate the absorption mechanism, a single-band absorber with one cross-shaped gold resonator was studied, as shown in Figure 4a. The thicknesses of the dielectric layer and the gold substrate are 220 and 200 nm, respectively. Figure 4b clearly shows that the length of the resonator strips has a significant impact on the resonant frequency; a red shift occurs when the length increases, suggesting that an absorber working at a specific frequency can be designed by adjusting the length of the strips. Figure 4c shows that the width of the resonator strips has little effect on the location of the absorption peaks. Therefore, it is possible to select a small width to realize high integration of devices. With symmetrical resonators, Figure 4d indicates that the absorption spectrum obtained by p-, s-, or circularly polarized incident waves have no significant differences.

**Figure 5:**
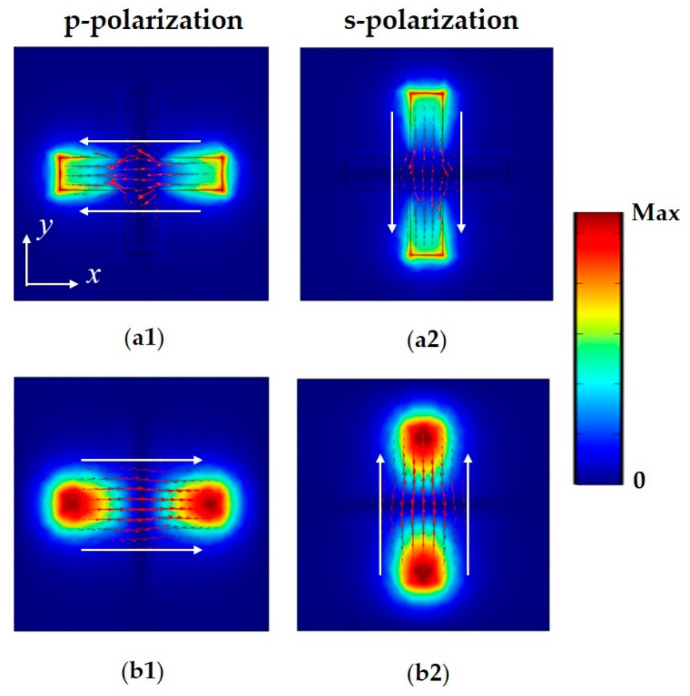
Distributions of the electric field amplitude and surface currents at the resonant wavelength on the cross-shaped metallic resonator for (**a1**) p-polarization and (**a2**) s-polarization, and the gold ground plane for (**b1**) p-polarization and (**b2**) s-polarization.

In Figure 5, the electric field amplitude and surface current distributions on the resonator and the ground plane are plotted. Evidently, the electric fields are localized near the left and right ends of the horizontal strip in the case of p-polarized wave incidence (Figure 5a1), which can be attributed to the accumulation of opposite charges at the ends of the metallic arm, resulting in electric dipole resonance. The strong coupling of the electric dipole results in a reverse charge distribution on the bottom plate. The electric field distribution at the corresponding location is shown in Figure 5b1. The surface currents on the top layer mainly flow along the negative *x*-axis direction, and those on the bottom flow in the opposite direction because of the reversed electric dipole moments. Consequently, a magnetic polariton (or “magnetic atom”) [30,31] is excited, which can induce a strong magnetic resonance and cause a resonant fall in the reflection spectrum [32]. For s-polarized wave incidence, the vertical strip is excited, forming an equivalent current loop along the *y*-axis. For both cases of wave incidence, the excited electromagnetic resonances confine and dissipate the electromagnetic energy in the absorber, resulting in perfect absorption close to 100%.

**Figure 6:**
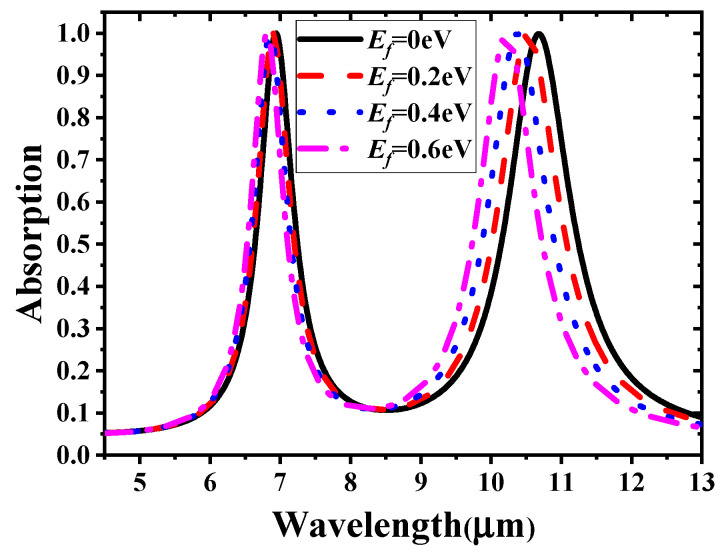
Variation in absorption spectrum with *E_f_*_1_ = *E_f_*_2_ ranging from 0 to 0.6 eV.

**Figure 7:**
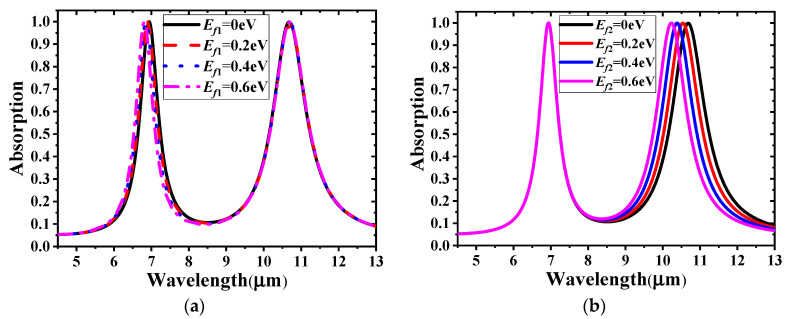
Absorption spectra by independently tuning (**a**) *E_f_*_1_ and (**b**) *E_f_*_2_.

The two absorption bands of the proposed GMA can be tuned as a whole or separately by different gate voltage loading methods on the two independent sets of graphene ribbons. As shown in Figure 6, the absorption spectrum blue shifts when the Fermi levels vary from 0 to 0.6 eV simultaneously. The first resonance peak shifts from 6.94 to 6.81 μm and the second one shifts from 10.68 to 10.22 μm; the absorption values of both peaks remain higher than 99%. To investigate the independent tuning of the two absorption bands, the Fermi level of one set of graphene ribbons was tuned, while that of the other was kept unchanged. The results are presented in Figure 7. Figure 7a shows that the first peak exhibits a slight blue shift when *E_f_*_1_ increases from 0 to 0.6 eV, and the second peak with *E_f_*_2_ = 0 remains unchanged, as expected. Figure 7b illustrates the absorption spectrum with constant *E_f_*_1_ and different *E_f_*_2_ values. Similar to the trend in the previous case, a blue shift evidently occurs for the second peak.

It is important to note that the tuning efficiencies for the two resonance peaks are significantly different under the same variation in the applied gate voltages loaded on graphene; the second resonant peak exhibits a better response. For instance, when *E_f_*_1_ and *E_f_*_2_ have the same increase from 0.4 to 0.6 eV, the absorption peak at the shorter wavelength shifts from 6.86 to 6.81 μm and that at the longer wavelength shifts from 10.37 to 10.22 μm. Assuming that the relative change in the resonant wavelengths is defined as *δ*_λ_ = 100 × ($\lambda_{R}^{1}$ − $\lambda_{R}^{2}$)/λ*_c_*, where $\lambda_{R}^{1}$ and $\lambda_{R}^{2}$ are the resonant wavelengths for *E_f_* = 0.4 eV and *E_f_* = 0.6 eV, respectively, and λ*_c_* = ($\lambda_{R}^{1}$ *+* $\lambda_{R}^{2}$)/2 [33], the spectral shifts can be quantified as 0.732% and 1.457%, respectively. This phenomenon can be explained by the perturbation theory of graphene on a metamaterial resonator. The change in the resonant frequency is given by [34]:

(3) $$\Delta \omega =\left(Im\left(\sigma_{g}\left(\omega \right)\right)-jRe\left(\sigma_{g}\left(\omega \right)\right)\right)\frac{\int_{S}{\left|E_{xy}\right|}^{2}dS}{W_{0}}$$

where *S* is the graphene area, *σ*_g_ (ω) denotes the graphene conductivity, $\left|E_{xy}\right|$ is the amplitude of the electric field on the plane of graphene, and *W*_0_ represents the stored electromagnetic energy in an uncovered metamaterial resonator. Thus, the spectral shift in the resonant frequency Re (∆ω) is related to Im (*σ*_g_(ω)), and the corresponding amplitude modulation of absorption, Im (∆ω), is determined by Re (*σ*_g_(ω)). The spectral shift is dominant over the amplitude modulation as Im (*σ*_g_(ω)) > Re (*σ*_g_(ω)), which is valid in the mid-infrared region [27,34]. The change in Im (σ_g_(ω)) increases with wavelength for the same change in *E_f_*, as shown in Figure 2b, explaining why the relative change in the resonant wavelength at the second resonance peak is greater than that at the first one.

**Figure 8:**
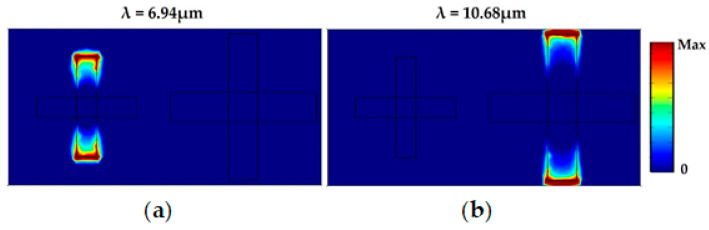
Amplitude of the electric field on the plane of graphene at the resonance peaks: (**a**) λ = 6.94 µm and (**b**) λ = 10.68 µm.

In order to interpret the resonance of the proposed absorber, the distributions of the electric field amplitude on the plane of graphene at resonant wavelengths were investigated. In Figure 8a, the electric field is distributed along the edge of the smaller cross-shaped resonator, which indicates that the smaller resonator is excited by the incident electromagnetic wave and the absorption peak at 6.94 μm is obtained. As shown in Figure 8b, the electric field is localized and concentrated at specific regions of the larger cross-shaped resonator. This illustrates that the perfect absorption at 10.68 μm is achieved due to electromagnetic resonance at the larger cross resonator.

**Figure 9:**
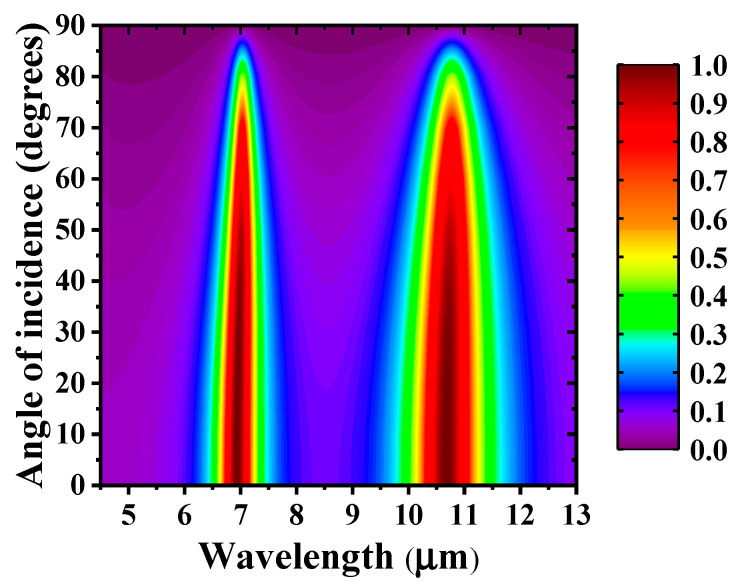
Absorption spectrum for s-polarized incident light as a function of wavelength and incident angle.

Figure 9 shows that the absorption spectrum changes with the incident angle of the s-polarized wave in the range of 0° to 90°. Both absorption bands maintain high absorption over a wide range of incident angles (up to 60°), indicating that the wide-angle nature of MA is unhindered.

**Figure 10:**
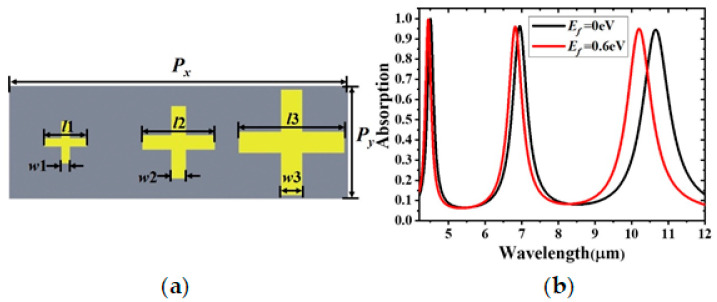
(**a**) Schematic diagram and (**b**) absorption spectrum of the triple-band absorber.

A triple-band absorber with three resonators of varied dimensions in the unit cell was designed, as shown in Figure 10a. The lengths of the strips are *l*1 = 1.4 μm, *l*2 = 2.4 μm, and *l*3 = 3.5 μm*,* and their widths are *w*i = 0.2 × *l*i (i = 1, 2, 3). The periods along the *x* and *y* directions are *P_x_* = 11.25 μm and *P_y_* = 3.75 μm, respectively. The other geometric parameters remain unchanged. In Figure 10b, three peaks are observed at 4.51, 6.95, and 10.66 μm, and their absorption rates are 0.998, 0.962, and 0.945, respectively. In earlier reports on electromagnetic MAs, the perfect absorption was explained in terms of matching the bulk metamaterial’s impedance $z=\sqrt{\mu_{eff}/\varepsilon_{eff}}$ to that of vacuum [4,7,35,36], where *ε_eff_* and *µ_eff_* are the effective permittivity and permeability of the bulk multi-layer metamaterial. To satisfy the condition of matched impedances, the condition of *ε_eff_* = *µ_eff_* is achieved by manipulating the spectral positions and strengths of the electric and magnetic resonances of the electromagnetic MAs [35]. The triple-band absorber consists of metal/dielectric-spacer/metal structure, allowing us to regulate absorption by varying the thickness of dielectric and, hence, *ε_eff_* and *µ_eff_*. The absorption peaks decrease slightly at 6.95 and 10.66 μm due to the mismatch between the dimensions of polytetrafluorethylene and the corresponding two resonators, resulting in an impedance mismatch between the structure and its surrounding [37]. However, the absorber is still efficient with an absorption greater than 90%. At the same time, the spectral shift of the absorption peaks increases with the resonant wavelengths because of the larger ΔIm (*σ*_g_) at longer wavelengths for the same ∆*E_f_*.

**Figure 11:**
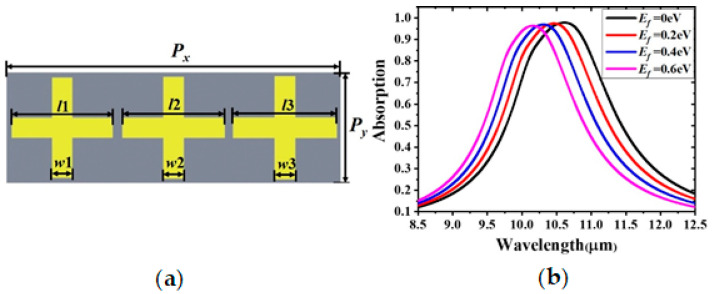
(**a**) Schematic diagram and (**b**) absorption spectrum of the broadband absorber.

Furthermore, a broadband absorber was designed using similarly sized resonators in the unit cell. As shown in Figure 11a, the length of the strips of the three gold cross resonators are *l*1 = 3.4 μm, *l*2 = 3.45 μm, and *l*3 = 3.5 μm, and their widths are *w*i = 0.2 × *l*i (i = 1, 2, 3). As depicted in Figure 11b, a composite wide-band absorption spectrum is obtained. As earlier, it blue shifts with the increasing Fermi level, ranging from 0 to 0.6 eV. Compared with the single-band absorber shown in Figure 4a, the full width at half maximum of the broadband absorption is increased by about 1.5 µm. Similarly, a number of multispectral MAs can be achieved by adding appropriate gold resonators. Therefore, the proposed structure provides a flexible model for multi-band and broadband absorbers.

## 4. Conclusions

In summary, an independently tunable multipurpose absorber with a single layer of metal-graphene metamaterials is proposed at mid-infrared frequencies. Compared with the previous work, the proposed dual-band absorber achieves independent tuning of two absorption peaks through reasonably designing the shape and arrangement of graphene and provides a flexible model for multi-band and broadband absorbers. The values of the absorption peaks are higher than 99%, and the structure does not hinder the wide-angle nature of MAs. The absorption spectrum is blue shifted when the Fermi level of graphene increases, and each resonance peak can be tuned independently. Triple-band and broadband absorbers were further achieved by appropriate design. The results of this study suggest that the proposed multipurpose structure can be widely used in light harvesting and thermal absorbing.

## Data Availability

The data presented in this study are available on request from the corresponding author.
